# Global cross-database search system for X-ray absorption spectra

**DOI:** 10.1107/S1600577525002206

**Published:** 2025-04-11

**Authors:** Masashi Ishii, Asahiko Matsuda, Koichi Sakamoto, Shohei Yamashita, Yasuhiro Niwa, Yasuhiro Inada

**Affiliations:** a Center for Basic Research on Materials National Institute for Materials Science (NIMS) 1-2-1 Sengen Tsukuba Ibaraki305-0047 Japan; b Materials Data Platform National Institute for Materials Science (NIMS) 1-1 Namiki Tsukuba Ibaraki305-0044 Japan; c Institute of Materials Structure Science High Energy Accelerator Research Organization (KEK) 1-1 Oho Tsukuba Ibaraki305-0801 Japan; d College of Life Sciences Ritsumeikan University 1-1-1 Noji-higashi Kusatsu Shiga525-8577 Japan; University College London, United Kingdom

**Keywords:** International XAFS DB portal, cross-database search, terminology, ontology, semantics

## Abstract

While the importance of a systematic overview of scientific data and demands toward data integration are increasing, data capitalization and confidentiality are also emerging competitively. X-ray absorption spectroscopy has a strong tradition of data sharing, as cross-referencing data enhances the detailed understanding of the obtained spectra. While physically integrating databases in various formats is impractical, a system has been successfully developed that allows cross-searching among Japanese, USA and European databases in cyberspace. This achievement is made possible through the realization of ‘vocabulary unification’ and ‘knowledge unification’ on a global scale, implemented in publicly accessible endpoints. This paper provides a summary of the concepts of terminology, ontology and semantics for X-ray spectroscopy behind this system, and presents a pilot case study along with future directions for data integration in synchrotron radiation science.

## Abstract 2

A portal to cross-search XAFS databases worldwide has been created and the unification of vocabulary and knowledge behind it are discussed.

## 1. Introduction

Currently, there are limited examples of global-scale integrated management for materials data, most of which are closely tied to industrial applications and are typically highly confidential. One notable example of effective integrated scientific data management is the Protein Data Bank (PDB) (PDB, 2024 ▸). The pioneering efforts in data sharing that began in an era when digitization seemed almost inconceivable have been thoroughly documented (Strasser, 2019 ▸); therefore, a detailed review is unnecessary here. Although systematically storing and sharing materials data following the PDB model poses significant challenges, this study has developed a practical approach for data sharing while protecting the rights of each data holder. This approach has been implemented as a web-accessible database portal (IXDB, 2024 ▸). The portal focuses on spectral data from a widely used synchrotron radiation technique, X-ray absorption fine structure (XAFS) (Kincaid & Eisenberger, 1975 ▸; Rehr & Albers, 2000 ▸). With this portal, XAFS users can search across databases in Japan, USA and Europe equally, and can immediately access these linked databases.

As a foundational work for this study, it is essential to reference the MDR XAFS DB project (Ishii *et al.*, 2021 ▸). XAFS is a synchrotron radiation experiment that precisely provides chemical bonding states and local structures at the atomic level. Since the atomic-level local information is inherently less confidential and the data interpretation can be enriched by comparing it with other spectra, there is a potential demand for data sharing. Under this background, the project MDR XAFS DB was launched in 2018 to aggregate XAFS data from leading synchrotron-radiation-related institutes in Japan and make it available as a database on the Materials Data Repository (MDR) of the National Institute for Materials Science (NIMS). Since the publication of a paper on this initiative (Ishii *et al.*, 2023 ▸), the number of participating institutions has expanded to six, and the database now provides access to 2263 XAFS spectra. The database features 56 unique absorption elements and 74 absorption edges. Importantly, the specimen names, which were not consistent when the data were provided, have been standardized through the NIMS Materials Vocabulary management system, MatVoc (MatVoc, 2024 ▸). This standardization ensures equal data findability, unifies accessibility, and eliminates institutional discrepancies.

The next goal is to extend this cross-search functionality globally, which will require addressing several challenges, starting with the international sharing of MatVoc. The target databases for this global cross-search include MDR XAFS DB, XASLIB in the USA (XASLIB, 2024 ▸), SSHADE/FAME (Kieffer & Testemale, 2024 ▸) and LISA XAS Database (Puri, 2024 ▸) in Europe. The number of spectra to be integrated into this worldwide framework includes 277 from XASLIB, 517 from SSHADE/FAME and 48 from LISA XAS Database; combined with the 2263 spectra from the MDR XAFS database, the total reaches 3104.

The web application developed for this cross-database search is henceforth referred to as the International XAFS Database Portal (IXDB). The practical construction policy of IXDB is as follows:

(i) Cross-searching all data across the four databases using two consistent terms — absorption edge and specimen name — that are uniformly included in XAFS measurement metadata.

(ii) To respect the rights and functions of each database, IXDB will only display links to the databases and their providing institutions, without hosting any data itself.

(iii) Data retrieval and processing is performed by external shared endpoints: create a framework that allows anyone to easily build a customized user interface (UI) and help increase data findability through economical portals.

## 2. Definition of academic requirements for IXDB

The academic requirements necessary for realization of IXDB are as follows:

(i) Introduction of ‘vocabulary unification’ to standardize database content (terminology).

(ii) Introduction of ‘knowledge unification’ to harmonize various data sources (ontology).

(iii) Development of protocols for sharing vocabulary and knowledge (semantics).

The requirements outlined here are not specific to IXDB alone, but can serve as general guidelines for systems designed for extensive data collaboration and utilization. The following subsections provide a detailed description of each of these requirements.

### 2.1. Vocabulary unification (terminology)

In recent data utilization platforms, attaching metadata to experimental data has become standard practice. It is essential to unify metadata items (keys) for reuse of experimental data with reproducibility and reliability (Ravel & Newville, 2016 ▸). For XAFS, unified metadata may encompass the operating conditions of the storage ring in synchrotron radiation facilities, the insertion device, the optical configuration of the monochromator, focusing and higher-order light elimination mirrors, and the detailed conditions of the signal detection system surrounding the specimen (XAFS Metadata Schema, 2024 ▸). On the other hand, as outlined in the construction policy, the only keys used for cross-database searches in IXDB are absorption edge and specimen name, both of which are consistently recorded in XAFS measurements. The limited keys indicate that the aim of such searches is to retrieve a broad array of spectra based on a few common conditions, rather than pinpointing a single spectrum with highly detailed conditions. The essential challenge here is not the unification of keys but rather the standardization of their descriptions (values). Specifically, specimen names must be standardized across institutions; otherwise, IXDB will function more like a traditional individual specimen search rather than an effective cross-database search. Vocabulary unification can be achieved through a dictionary that maps various local specimen names to unique material names.

### 2.2. Knowledge unification (ontology)

Although there are variations in database services, when dealing with XAFS spectral data, there should be a common physical concept, referred to as ‘XAFS knowledge’, that is independent of the service styles. This XAFS knowledge can be made ontologically machine-understandable. Obviously, it is not easy to rigorously describe the XAFS knowledge involved in X-ray excitation process. The knowledge here does not treat XAFS as a word in natural language, but rather means scientifically relating it to other vocabularies. In other words, it states that the absorption edge is determined by the element to be excited and the electron to be excited. From a materials science perspective, it also defines that there is an excited element in the target sample and that there are excited electrons within it. Strictly speaking, we would also need to define dynamic states during the excitation, and this would be useful for advanced measurements and spectral interpretation. However, such advanced concepts are not useful for the cross-searching dealt with in this article. Therefore, it is sufficient to set ontological constraints based on vocabulary linkages described above. This machine-readable notation is explained in detail in Section 3. Additionally, there is attribution information (hereafter referred to as the ‘attribution knowledge’) that is crucial for data reuse, such as the URL to the spectrum and the name of the data holder. Practically, when selecting from a list of spectra, one may consider the strengths and limitations of the data-providing institutions, such as the photon energy range of intense X-rays from their storage ring, which is generally known even if not explicitly stated in the metadata. While the URL linking to the spectrum is not experimental data itself, it is essential for data access. We refer to the combination of XAFS knowledge and attribution knowledge as ‘dataset knowledge’. Regardless of whether the database is MDR XAFS DB or another, dataset knowledge should be uniquely determined. This uniqueness enables universal cross-search across databases using a single algorithm.

### 2.3. Sharing vocabulary and knowledge (semantics)

**Figure 1:**
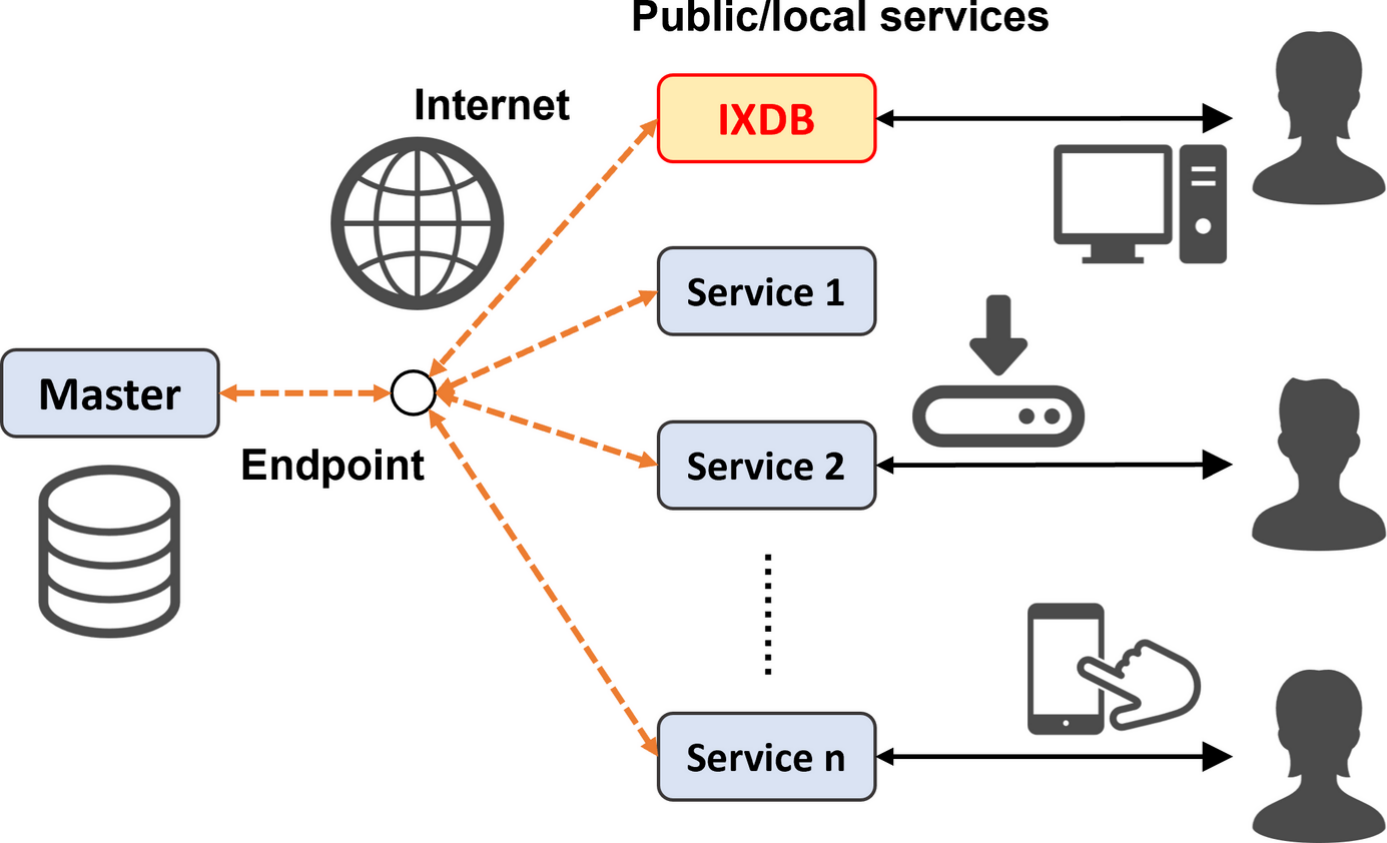
A mechanism for sharing master data on a worldwide scale. The database is managed in one place, and each service can design its services economically.

The vocabularies and knowledge discussed in Sections 2.1 and 2.2 must be shared and interoperable across various systems. Additionally, version forking of the actual data (instances) must be avoided. Ideally, both vocabulary and knowledge should be centralized in a master data endpoint that anyone can refer to, with updates from this endpoint being immediately reflected in all linked services, including IXDB. Once such endpoints are established, the connected service systems will operate as an economical and reliable network, always providing consistent data. More specifically, this means that a database (the ‘master’ in Fig. 1 ▸) is available on the Internet, accepting queries written in a semantic language and returning appropriate responses. Additionally, there is an open port (the ‘endpoint’ in Fig. 1 ▸) to receive these queries. In modern databases, queries are typically sent from the UI to the main database within a closed system through an application programming interface (API), with the results displayed on the UI. Now, considering that the database and UI are spatially separated and both are located on the Internet, the mechanism in Fig. 1 ▸ is easier to understand. Since all UIs on the Internet (the ‘services’ in Fig. 1 ▸) can access the common ‘master’ database and retrieve the original data at any time, there is no risk of data discrepancies. Consequently, IXDB functions as one such service, allowing multiple users to access data through semantic conversations via endpoints, which minimizes data management costs. While data sharing is often equated with publishing data on a public server, the mechanism illustrated in Fig. 1 ▸ represents what we consider true data sharing.

## 3. Implementation and discussion

This section describes the following three implementations corresponding to the requirements defined in Section 2 and the considerations obtained from them.

(i) XAFS dictionary creation.

(ii) XAFS knowledge creation.

(iii) Portal creation.

### 3.1. XAFS dictionary creation

As discussed in Section 2.1, the role of the dictionary is to provide unique material names for various local specimen name inputs. For machine readability, unique material names are managed using IDs rather than text. A well known machine-readable material ID is the CAS registry number. Although the CAS number is widely used, the difficulty in nomenclature for different instances may result in multiple IDs for a single material, some of which have been reorganized or integrated over time. For example, the CAS numbers for FeOOH, which has polymorphic forms, are 1310-14-1 and 20344-49-4, both of which have had their numbers deleted or replaced in the past. This variability indicates that substance identification can differ across domains, highlighting the need for a dictionary specific to XAFS. Consequently, we have strengthened the NIMS XAFS DB Project Materials Dictionary within MatVoc (MatVoc, 2024 ▸), which was developed as part of the domestic initiative described in Section 1 for international cross-database search (hereafter referred to as the ‘XAFS dictionary’). In MatVoc, vocabulary registration involves automatic assignment of a vocabulary ID (‘QID’ provided by Q + number) categorized in the XAFS dictionary when a set of representative names (labels), definitions (descriptions) and broader categories (upper classes) are registered. Synonyms can also be registered, enhancing the system’s robustness against variations in search terms. At present, the XAFS dictionary contains 955 material names and over 9100 synonyms. Note that these vocabularies are defined within the MatVoc namespace (https://matvoc.nims.go.jp/entity/) and have shareable URIs (uniform resource identifiers),*i.e.* global IDs that can be accessed from anywhere. These URIs are used for semantic database searches in IXDB.

The XAFS dictionary produces closed lexical space in MatVoc with the following four classes: Chemicals (Q2828), X-ray absorption edge (Q2487), Element (Q2392) and Electron (Q2823). For instance, ‘Chemicals (Q2828)’ indicates that the class is identified by the QID (Q2828) and is represented by the name ‘Chemicals’. This nomenclature allows us to view a conceptual hierarchy. The actual hierarchy is considerably more detailed; material names are subclasses of Chemicals (Q2828), and X-ray absorption edges are subclasses of X-ray absorption edge (Q2487). The implementation of these lower classes will be discussed in Sections 3.1.1 and 3.1.2, respectively.

#### 3.1.1. Implementation of lower classes for Chemicals (Q2828)

**Figure 2:**
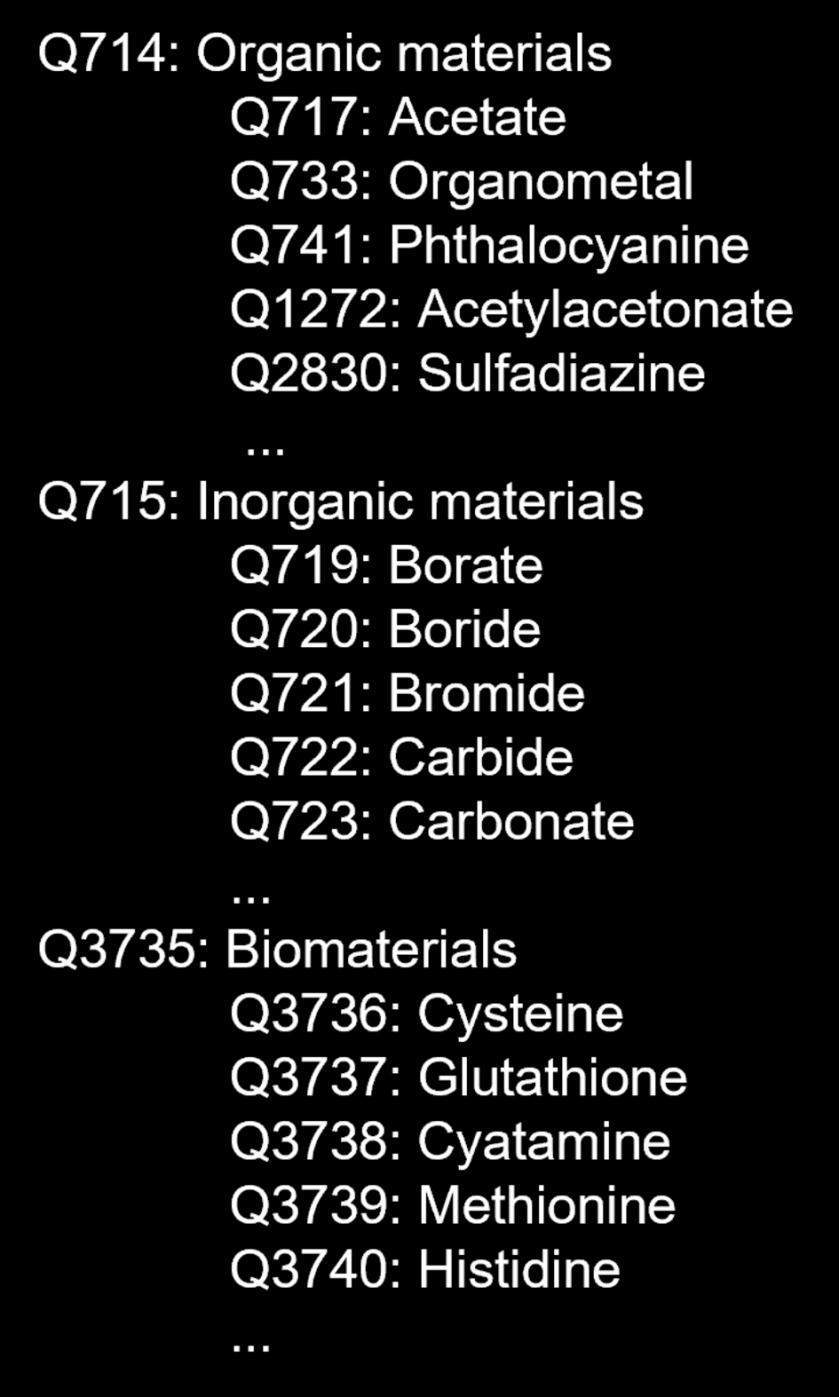
Three subclasses under Q2828: Chemicals. Q714: Organic materials; Q715: Inorganic materials; Q3735: Biomaterials. Representative sub­classes in each hierarchy are also shown.

The three subclasses under Q2828 are Organic materials (Q714), Inorganic materials (Q715) and Biomaterials (Q3735). Fig. 2 ▸ illustrates a schematic diagram of the chemical hierarchy, including representative subclasses for each category. For the complete hierarchy, refer to MatVoc (https://matvoc.nims.go.jp/explore/en/results/Q2828). In particular, organic materials (Q714) are often restricted to organic compounds containing heavy metals that can be excited by hard X-rays, meaning its subclasses do not cover the general organic chemistry. Despite this limitation, our strategy is to allow creation of materials dictionary only for XAFS, and to explicitly link (bridge) to other dictionaries that cover the entire range of materials. In fact, we previously reported (Ishii *et al.*, 2023 ▸) that a bridge to PubChem, a comprehensive low-molecular-weight substance dictionary, can be established using skos:closeMatch. Here, skos is the Simple Knowledge Organization System, where predicates for describing a body of knowledge are compiled by W3C (SKOS, 2024 ▸). For more details, the definition is shown at https://www.w3.org/2004/02/skos/core#closeMatch.

Even for Inorganic materials (Q715), which have relatively simple and comprehensive molecular structures, the hierarchical structure of XAFS often does not align with the macroscopic structure of the material. Specifically, when observing impurities in bulk, XAFS, which is sensitive to local structures, focuses on the impurity rather than the extensive bulk surrounding it. For example, ‘Mouse lung exposed to CeO_2_ particles’ in SSHADE/FAME (Chaurand & Collin, 2017 ▸) would, from a medical point of view, be in the category of ‘mouse lung’ or ‘lung’. However, XAFS is focused on CeO_2_, specifically on the local structure of cerium within it. Consequently, it does not identify the lungs as an organ but assesses the detection capability in measuring the distribution of CeO_2_ in the lungs (Chaurand *et al.*, 2018 ▸). In fact, no other lung data are available in the databases beyond this specimen, rendering cross-database searches ineffective for this particular context. On the other hand, 11 CeO_2_-related spectra, which is the target of the cross-search, are provided from 7 different databases. Clearly, the XAFS perspective necessitates a different approach to cross-database search compared with the medical perspective. Here are a few examples. When examining the Co absorption edge of cobalt-doped aluminium oxide (Q3785) using XAFS (Vichery & Maurin, 2012 ▸), the local structure around cobalt may be classified as a metal oxide complex like cobaltate, Oxide_ate (Q736), despite Al_2_O_3_ being an oxide. In contrast, when analyzing chondrites (*e.g.* Garenne *et al.*, 2013 ▸), the primary focus is on component ratios rather than local structures like additives, leveraging the high transmissivity of X-rays (Garenne *et al.*, 2019 ▸). In this scenario, having an astronomical category, such as Carbonaceous chondrite (Q3744), is advantageous. For instance, 57 carbonaceous chondrite specimens constitute a sufficient category for cross-database searches in XAFS. Ultimately, dictionaries must be tailored to the research purpose and are not universally applicable. Of course, as with organic compounds, it may be possible to achieve a universal material classification by linking through skos:closeMatch. It is important to recognize that a purpose-oriented dictionary, rather than a general one, will facilitate cross-database searches for XAFS spectra.

#### 3.1.2. Implementation of lower classes for X-ray absorption edge (Q2487)

**Figure 3:**
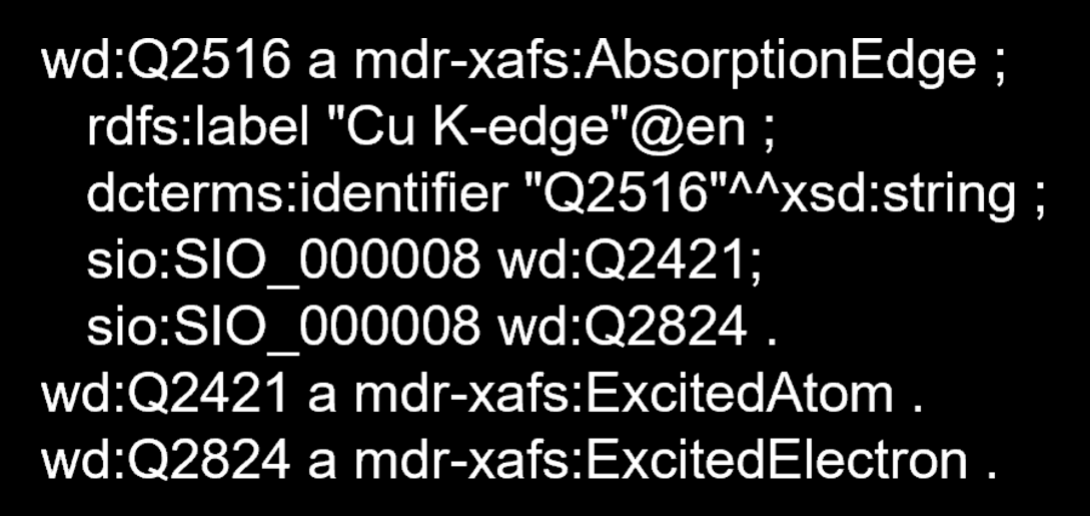
An example of a machine-readable description of an absorption edge (Q2516: Cu *K*-edge).

**Figure 4:**
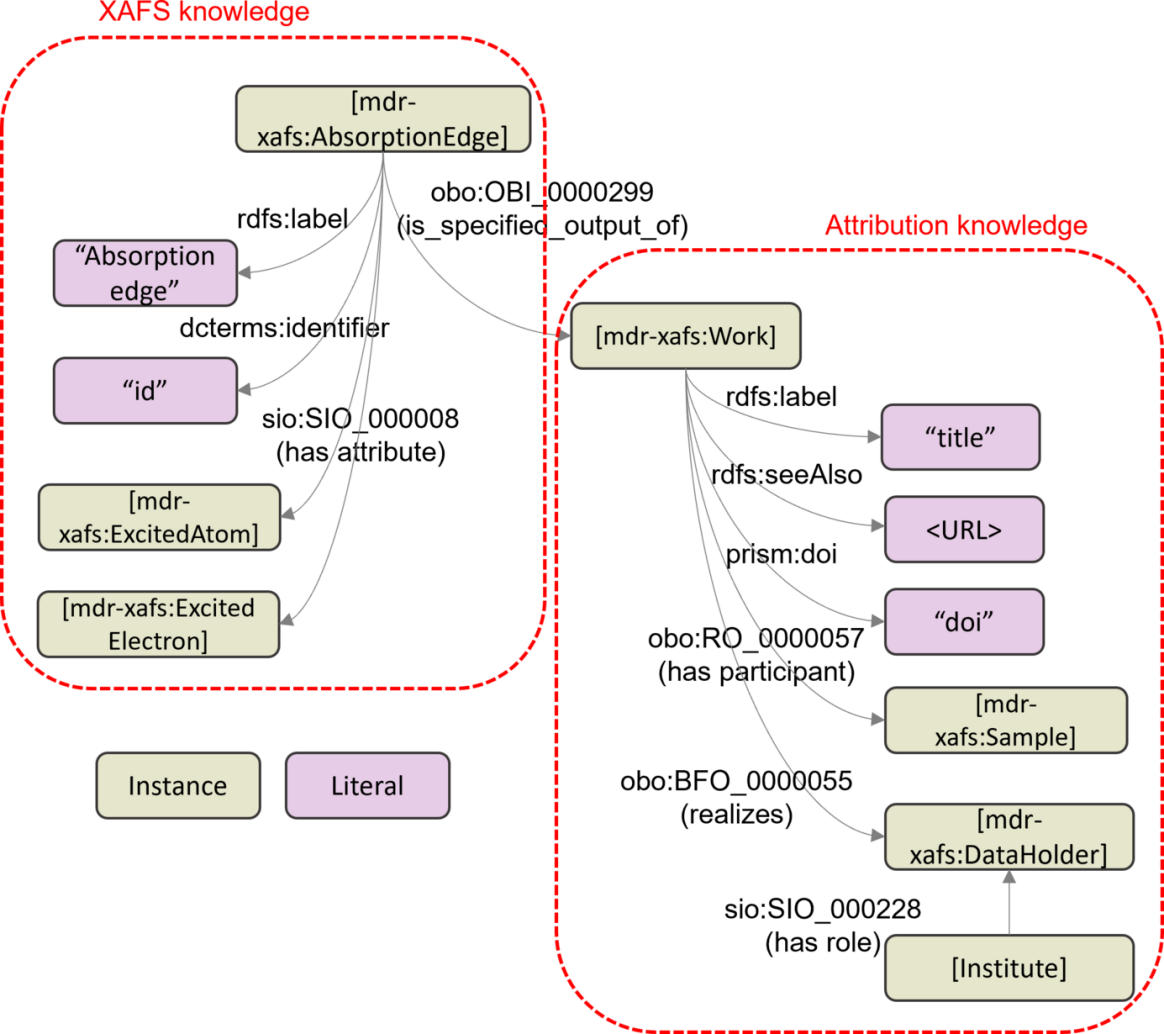
Directed graph structure of ‘knowledge’ combining ‘XAFS knowledge’ (left side) and ‘attribute knowledge’ (right side).

In the constructed XAFS dictionary, absorption edges are directly categorized under Q2487 and encompass the *K*-edge and *L*-edge, spanning from hydrogen to plutonium. For instance, the Cu *K*-edge, a standard in XAFS, is defined by Q2516. It is important to note that an absorption edge is defined by the combination of an absorption element and an inner-shell electron, with these vocabularies being classified as Element (Q2392) and Electron (Q2823), respectively (see the four hierarchical categories outlined in Section 3.1). Although the absorption edges are fewer in number compared with material names, the key objective is to establish machine-readable descriptions that link absorption edges with electronic states, thereby integrating XAFS spectra into broader physical knowledge. Fig. 3 ▸ illustrates the machine-readable description (schema) applied to the Cu *K*-edge (Q2516), formatted according to the internationally standardized Resource Description Framework (RDF) (RDF, 2024 ▸). RDF is a semantic data representation framework that depicts information as subject–predicate–object combinations, known as ‘triples’. A visual representation of the schema using a directed graph is shown in Fig. 4 ▸ of Section 3.2, which includes human-readable labels to enhance understanding of the predicates in these triples. For non-experts in semantics, the schema in Fig. 3 ▸ is explained as follows. It asserts that Q2516 (Cu *K*-edge) has attributes Q2421 (excited element: Cu) and Q2824 (excited electron: *K*-shell electron), with ‘absorption edge’, ‘excited element’ and ‘excited electron’ being defined in the MDR-XAFS ontology under the following namespace: https://dice.nims.go.jp/ontology/mdr-xafs-ont/Schema#. For example, for the absorption edge (mdr-xafs:AbsorptionEdge), the definition can be found at the following web-accessible URI: https://dice.nims.go.jp/ontology/mdr-xafs-ont/Schema#AbsorptionEdge. As will be shown later, the general physical knowledge of inner-shell excitations provides a data link between XAFS and other synchrotron radiation experiments.

### 3.2. Creation of XAFS common knowledge

The dictionary discussed in Section 3.1 can be used to convert vocabularies from human-readable to machine-readable expressions. Given that databases have varying table structures and search algorithms, achieving cross-database search requires not only a unified vocabulary QID but also unified dataset knowledge. As described in Fig. 3 ▸, the representation of knowledge using RDF (‘triples’) can be illustrated mathematically as a directed graph. In other words, if the subject and object in a triple are represented as nodes and connected by a predicate as an edge, a chain of data can be expressed, and ultimately knowledge can be constructed. Just as arbitrary nodes and edges can be manipulated in graph theory, arbitrary information can be extracted from this knowledge graph. Fig. 4 ▸ illustrates the directed graph structure of the knowledge designed in this study; XAFS knowledge and attribute knowledge, as described in Section 2.2, are displayed on the left and right sides of the figure, respectively. The key points for knowledge design are as follows:

(i) We define the XAFS dataset creation process as ‘Work’. Here, both XAFS and attribution knowledge are associated with the Work.

(ii) In the attribution knowledge, URL, DOI and data holders are specified.

(iii) In this Work, the specimen is referred to as a ‘participant’ to emphasize that it is central to the study, rather than merely an object to be measured. This distinction clarifies that the primary aim of this Work is not the development of instruments or similar aspects.

(iv) Several roles are required to conduct the Work, one of which is the data holder role. This role is fulfilled by the participating institution.

(v) The absorption edge is an outcome of the Work, and, as explained in Section 3.1.2, the XAFS knowledge specifying the absorption edge includes two attributes: the absorption element and the inner-shell electron. Therefore, this figure incorporates Fig. 3 ▸, which is represented using a directed graph.

This dataset knowledge meets the requirements necessary for cross-database search and is applicable to any XAFS database. The primary goal of introducing dataset knowledge is to enable batch searches of this unified structure using a single query based on the SPARQL Protocol and RDF Query Language (SPARQL) (SPARQL, 2024 ▸). This SPARQL query plays the role of extracting information from the directed graph mentioned above. This semantic schema can be expanded later without changing the query. For example, the crystal structure can be used as an attribute of the specimen. The ontological linkage with other vocabularies (ExPaNDS, 2024 ▸) will ultimately lead to the construction of a large scientific knowledge base.

### 3.3. Creation of the portal

**Figure 5:**
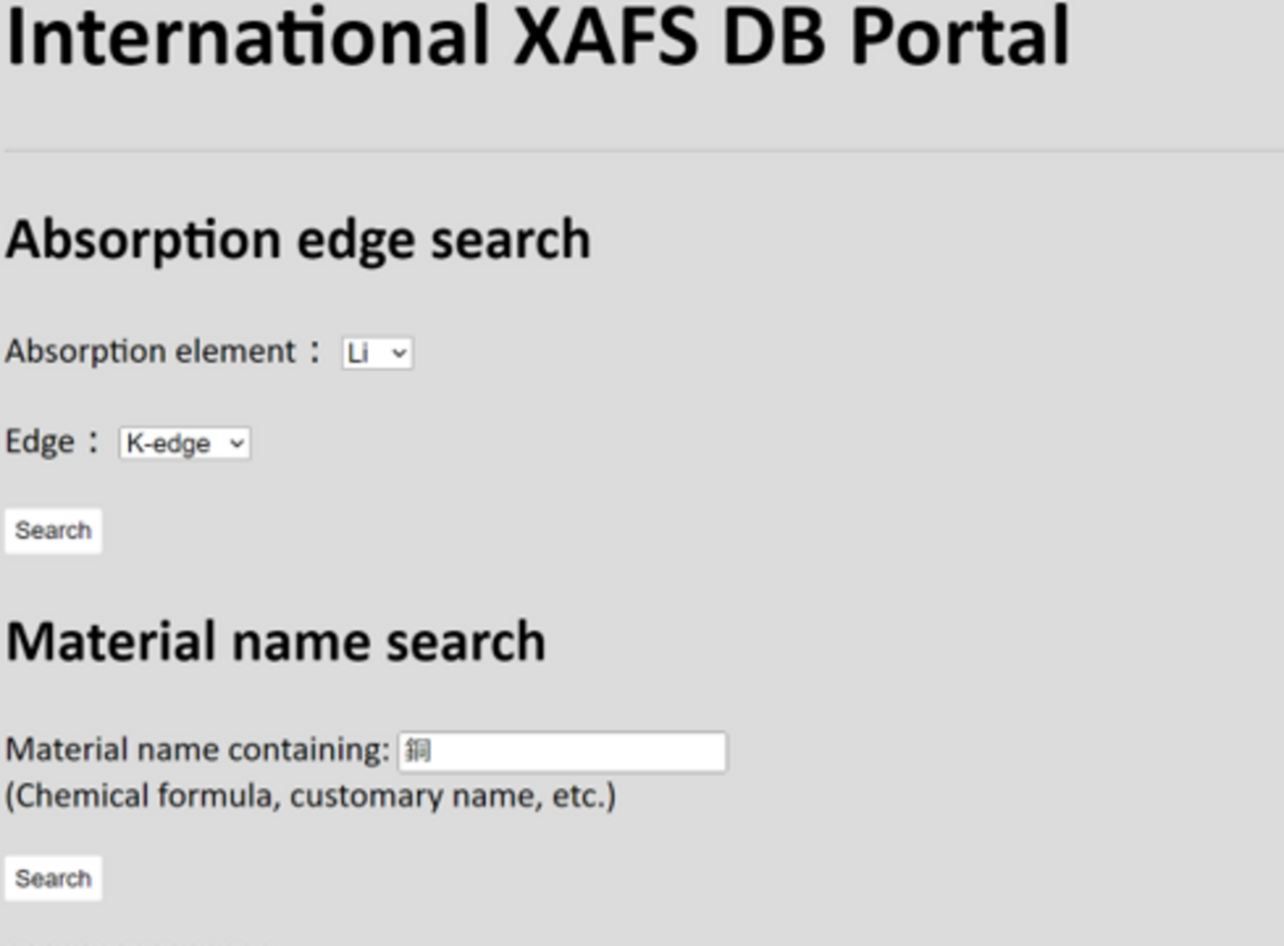
International XAFS DB Portal as a service of Japanese XAFS Society (https://ixdb.jxafs.org/).

The IXDB portal has a straightforward yet practical role: providing links to databases worldwide. It is built on a Python-based application that enables anyone, anywhere, to quickly create similar services (see Fig. 1 ▸). Currently, IXDB is accessible to the public as an official service under the sub-domain of the Japanese XAFS Society: https://ixdb.jxafs.org/. The main page is shown in Fig. 5 ▸. Users can perform cross-database searches by material name and absorption edge. Search results display the corresponding QID and representative name. By selecting the desired material name, users can obtain a URL link to the databases holding the data. This screen transition is familiar to XAFS users, ensuring that they will not experience confusion during operation. Since MatVoc includes Japanese synonyms, it is possible to search XAFS databases in Europe and USA using Japanese terms. Although this serves as a symbolic demonstration of synonym search capability, cross-database searches are also feasible with English common names and chemical formulas. While an ideal solution would be a collaborative editing wiki system to enrich synonyms, its implementation remains a future challenge, given that the purpose-oriented dictionary discussed in Section 3.1 also requires control over polysemy.

### 3.4. Maintaining and expanding the portal

While the services connected to the endpoints will no longer require data maintenance, managing the vocabulary and knowledge as a master resource will still be necessary. Particularly, when aiming to extend cross-database searches beyond the four databases currently implemented to include others worldwide, substantial effort will be required.

Specifically, the primary tasks will involve merging the names of measured samples, assigning new QIDs to new materials in MatVoc, and associating them with absorption edges. For instance, this process was completed in about a month for roughly 500 spectra from SSHADE/FAME. Ideally, this work could be carried out collaboratively by the community. To support this effort, a MatVoc wiki system is needed, along with clearly defined rules and guidelines. Our goal is to develop a collaborative system with the community to strengthen the data infrastructure.

## 4. Operation procedure of IXDB

Finally, we summarize the actual search flow in IXDB. The search flow consists of the following three steps:

(1) Machine-readable conversion of vocabulary (QID conversion).

(2) Cross-database search using knowledge.

(3) Indication of links to datasets and their data holders.

**Figure 6:**
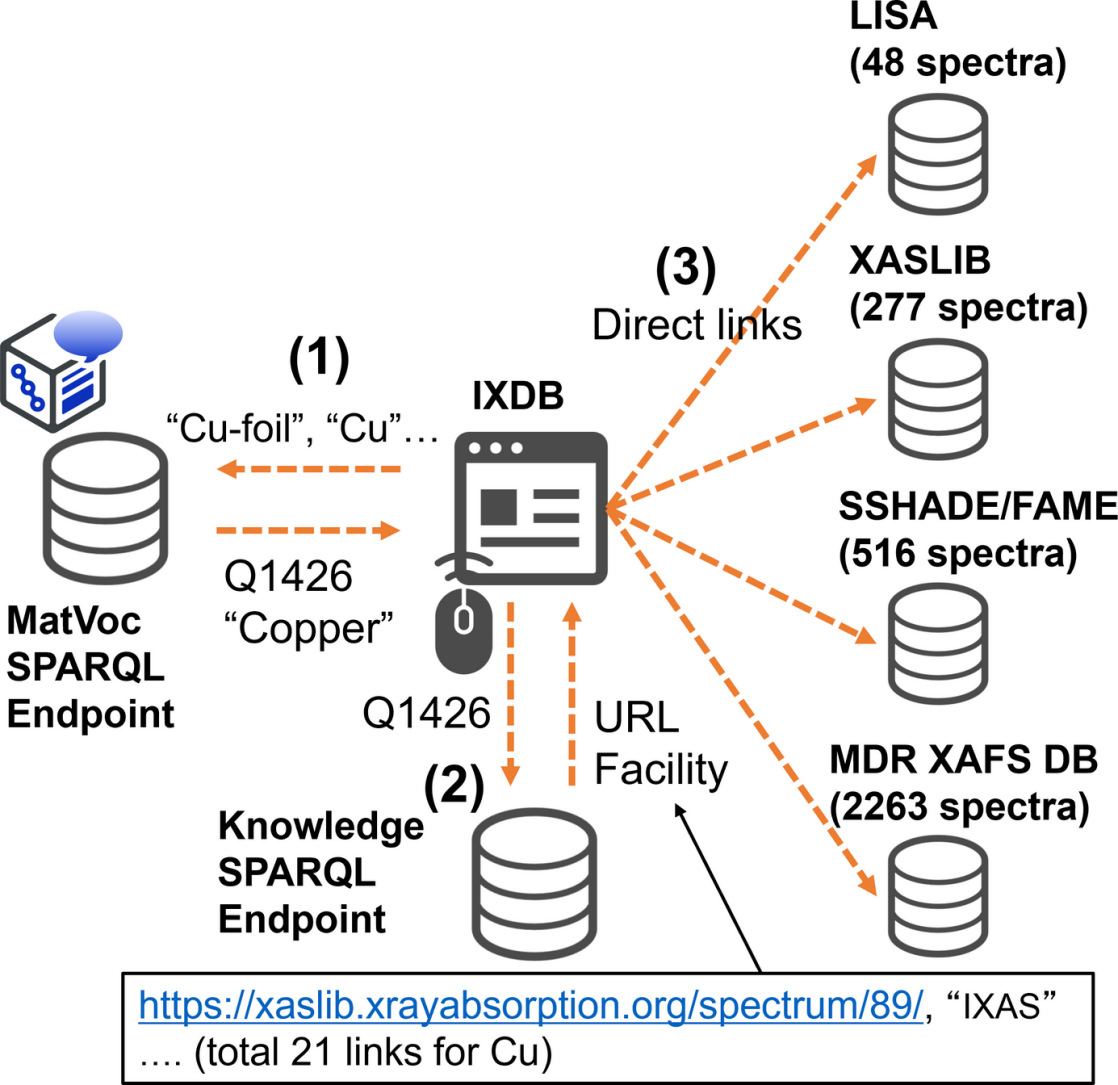
Schematic diagram of the IXDB system including query-and-responses flows.

Fig. 6 ▸ is a schematic diagram of the system including query-and-responses. The numbers in the figure correspond to the numbers above steps. The principle of each process is described below.

(1) Machine-readable conversion of vocabulary (QID conversion). In many cases, different specimen names are used across databases. For example, ‘Cobalt-iron oxide’ in MDR XAFS DB corresponds to ‘CoFe_2_O_4_’ in SSHADE/FAME, ‘Sodium selenate’ in SSHADE/FAME is the same as ‘Na_2_SeO_3_’ in XASLIB, and ‘Pyrolusite’ in XASLIB is equivalent to ‘Manganese (IV) oxide’ in MDR XAFS DB. Even though it should be possible to search using any term, when individual databases are used separately, human knowledge is required to recognize that they are the same material. Advanced databases like SSHADE/FAME have internal synonym dictionaries to manage variations in search terms, but for the international cross-database search implemented here the created XAFS dictionary is shared among all target databases. This conversion returns the representative name of the material to IXDB along with its QID for visibility, though the QID is the primary reference used in subsequent searches. In this example shown in Fig. 6 ▸, the representative name is ‘Copper’ and the QID is Q1426. The MatVoc SPARQL endpoint used for this QID conversion is: https://matvoc.nims.go.jp/graph/sparql. Appendix *A*[App appa] gives an example of the curl (client for URL) command including SPARQL that obtains the representative name and corresponding QID for chemical formulars containing ‘Cu’.

(2) Cross-database search using ‘knowledge’. Once a QID is assigned, as described in (1), the SPARQL query used to search for knowledge is consistent across all databases, facilitating cross-database searches. In this study, we customized the SPARQL to return both the name of the data holder and links to the data for each QID. The developed knowledge including the MDR-XAFS ontology is stored on the NIMS public SPARQL endpoint, which is https://materials-open-rdf.nims.go.jp/sparql. Appendix *B*[App appb] gives an example of the curl command including SPARQL that actually obtains the name of the data holder and links to the data for the Cu *K*-edge of copper. In essence, although all of the query descriptions here are represented using QIDs, the contents describe the procedure to retrieve information by tracing the directed graph in Fig. 4 ▸. Specifically, the steps are as follows: (*a*) Select Works associated with the normalized name of copper (Q1426). (*b*) These Works should output the absorption edge, where the excited atom is copper (Q2421), and the *K*-shell electron (Q2824) is excited. (*c*) Finally, the data holder for each Work and the corresponding URLs where the specific data are located should be indicated. This process enables the display of 21 links to spectra from all the databases involved in this project on the IXDB portal.

This procedure can be adapted to derive the material name by specifying a different absorption edge, such as the *L*_3_-edge (Q2599), or to generate a list of data provided by a specific data holder. This is a key advantage of semantic data representation, which allows the user to obtain the desired information as though engaging in the semantic conversation with the endpoint. The high degree of flexibility in designing SPARQL queries allows IXDB to incorporate additional functionalities. By generalizing the knowledge to inner-shell excitations, other measurement methods with similar physical processes to XAFS can be integrated into the same system for cross-database searches. For instance, IXDB currently demonstrates linkage with Hard X-ray Photoelectron Spectroscopy (HAXPES).

(3) Indication of links and data holding institutions. As a result of (2), IXDB provides links to spectra based on the user’s input of material name or absorption edge. For example, if the material name is specified as ‘silver’ (Q1292) alone, a list of 12 data holders and links will be displayed. If only the absorption edge is specified as ‘Fe *K*-edge’ (Q2513), a list of 291 spectrum data holders and links will be obtained. These links direct users to the relevant databases, with IXDB’s role being solely to make the data discoverable, while the individual databases manage the remaining functions related to data utilization. Users can access the data only after being redirected to the individual databases, ensuring that IXDB does not infringe upon the rights of data holders. Moreover, it should be emphasized that (1) and (2) utilize endpoints external to IXDB, with IXDB responsible only for managing data linkage. Consequently, IXDB functions as a cost-effective system without database capabilities. The clear segmentation of roles and the complete systemic and spatial separation of ID conversion, search and display functions means that not only IXDB but also other portals can be built and operated by anyone anywhere, and can be operated simultaneously. Provided that the query design in the portals is accurate, any portal will display the most current information without discrepancies. For example, a service that has been reorganized from the perspective of X-ray photoelectron spectroscopy (XPS) rather than XAFS is being released on a trial basis at https://materiage.org/. Detailed specifications of the endpoints will be published in the MDR.

## 5. Summary and prospects

The International XAFS DB (IXDB) portal for global cross-database searches in X-ray absorption spectroscopy has been developed and made publicly accessible. This portal has achieved unification of vocabulary and knowledge, addressing variations in search terms and normalizing database structures, thus facilitating the findability of all XAFS data. The knowledge developed is machine-understandable for inner-shell excitation and can be extended to other synchrotron radiation data. Notably, IXDB has implemented cross-database searches for XAFS and HAXPES. This strongly indicates the potential for integrating searches across synchrotron radiation data and other measurement data by further extending the unifications of vocabulary and knowledge.

## Data Availability

The data for the implemented application, vocabulary dictionary and knowledge base can be accessed via the URLs given in the paper.

## Appendix A An example of vocabulary unification using the MatVoc endpoint

An example of a curl command including SPARQL that obtains the representative name and corresponding QID for chemical formulars containing ‘Cu’. Line breaks have been inserted into the SPARQL for visibility. SPARQL can also be submitted from an online editor at https://matvoc.nims.go.jp/query/.[Chem scheme1]

## Appendix B An example of semantic conversation using dataset knowledge via NIMS public endpoint

An example of a curl command including SPARQL that actually obtains the name of the data holder and links to the data for the Cu *K*-edge of copper. Line breaks have been inserted into the SPARQL for visibility. SPARQL can also be submitted from an online editor at https://materials-open-rdf.nims.go.jp/sparql/.[Chem scheme2]
